# Basic Send-on-Delta Sampling for Signal Tracking-Error Reduction

**DOI:** 10.3390/s17020312

**Published:** 2017-02-08

**Authors:** Miguel Diaz-Cacho, Emma Delgado, Antonio Barreiro, Pablo Falcón

**Affiliations:** 1 School of Computer Engineering, Campus de Ourense, Edificio Politecnico, 32004 Ourense, Spain; 2 School of Industrial Engineering, Campus Lagoas-Marcosende, 36310 Vigo, Spain; emmad@uvigo.es (E.D.); abarreiro@uvigo.es (A.B.); pfalcon@uvigo.es (P.F.)

**Keywords:** Send-on-Delta, deadband, tracking error, networks

## Abstract

This paper investigates the dynamic selection of an appropriate threshold for basic Send-on-Delta (SoD) sampling strategies, given an available transmission rate to reduce the signal tracking-error. The paper formulates the error-reduction principle and proposes an algorithm that calculates, in real time, the amplitude threshold value (also called delta value) for a desired mean transmission rate. The algorithm is implemented to be computed in a Send-on-Delta driver and is tested with three signals that match the step response of a second order control system. Comparison results with a conformant periodic transmission strategy reveals that it improves deeply the tracking-error while maintaining the desired average throughput.

## 1. Introduction

New paradigms in sensors and actuators data transmission strategies are topics of interest due to the challenges that the increase in the amount of data presents in a completely inter-communicated world. Cloud computing, Internet of Things (IoT), sensor networks, multimedia, teleoperation, smart-phones and inter-vehicular communications are only a few examples of such a challenge. The most used transmission strategy is the periodic sampling transmission strategy, where sensor or controller data are periodically sent across networks and the parameter to be adjusted is the transmission period. The motivation for this periodic sampling nature is that it is easy to be implemented in computing environments, and there is a well known system theory around it.

On the other hand, amplitude sampling has grown in importance due its efficiency selecting the data to be processed or to be sent across the networks. Basic amplitude sampling is focused on transmitting a data packet when the value of the data deviates from a reference piece of data by a known value called delta value, deadband or threshold. The utility of this sampling strategy is proved by the implementation in real commercial sensors, like the EnOcean sensors [1], and its use is motivated because it allows for sending much less data, maintaining a similar signal tracking-error performance.

The amplitude sampling technique is mentioned in the literature as the “*send-on-delta*“ method [2], “*deadband*“ method [3], “*event-triggering*“, “*event-sampling*“ [4] or even “*Lebesque sampling*“ [5]. From now on, we refer to this strategy as the send-on-delta (SoD) method. The most common SoD method uses a constant delta value (*δ*), and the reference data is the last sent one ($x_{ls}$); this SoD strategy is called *last-sent SoD* or *basic SoD* and may be modeled by a send function S(t) like:

(1) $$S\left(t\right)=\left\{\begin{array}{c} 1\;\mathrm{if}\;\left||x_{ls}-x\left(t\right)|\right|\geq \delta ,\\ 0\;\mathrm{if}\;\left||x_{ls}-x\left(t\right)|\right|<\delta , \end{array}\right.$$

where x(t) is the candidate data to be sent at instant *t*. In place of using the send function represented in expression (1), some authors [6] use the function $∥x_{ls}-x\left(t\right)∥-\delta$ and employ the term *trigger-function*.

Apart from the basic SoD technique, there are some others, like the *relative deadband*, presented in [7], inspired by Weber’s law in human sensibility, where the deadband value is proportional to the value of the last-sent data ($\delta =c\cdot x_{ls}$); the *network-based deadband* [8,9] where the deadband value is selected depending on the network status; the *linear-predicted-data SoD* [10] where the reference data is a linear prediction calculated using the last-sent-data and the previous sampled data. Some other SoD strategies are focused on the trigger function, for example the *IAE-based SoD*, where the driver sends a sample if the sampling integral absolute error (IAE) is bigger than the deadband value [11], or the *Energy-error based SoD*, where a data is sent if the energy of the error measured in the last sampling interval is bigger than the deadband threshold [12]. In parallel, the SoD strategies are used to improve collaborative challenges through data networks [13,14].

Periodic-sampling and Send-on-Delta sampling are not the only solutions to deal with the data selection to be sent across the network. In the literature, we also find the ’*adaptive sampling*“ solutions. As commented in [15], send-on-delta solutions differ from the adaptive-sampling in the way that both selects the send instant; adaptive-sampling solutions does not monitor the data to be sent and compares it with others (as done in the SoD solutions), but instead, the data send instant is determined by a timer that is excited when an event occurs. The difference between these two transmission paradigms may be found in the scientific literature under the more technical terms ”event-triggering“ (which refers to the SoD solutions) and ”self-triggering“ (referred to the adaptive-sampling). A complete compilation about event-based sampling is included in the book [16].

The advantages of using SoD are that it may save network resources maintaining an acceptable application performance. Other resources saved by the SoD strategy are the battery consumption in wireless sensor networks or the CPU load. Besides the savings on network consumption, the SoD strategies may decrease the signal tracking-error [17,18], the system-error of control systems [4] or the estimation-error if an estimator is used in networked control systems [19]; this would be achieved if the average transmission rate remains the same as the transmission rate of a conforming periodic sampling strategy [18]. An example of such a situation would occur in 3G/4G commercial data communications, where the network operators sell an amount of data (*D*) to be sent monthly, which makes, in practical implementations, a mean available bandwidth. In this case, the conforming periodic sampling strategy would have a constant sampling rate of $\frac{D\cdot 8}{30\cdot 24\cdot 3600}$ bits/s, but the peak bandwidth is much bigger (until 100 Mbps in 4G networks). The SoD sampling strategy may be used to try to reduce significantly the signal tracking error, but maintaining the goal to send *D* bytes in one month.

Nevertheless, it is difficult to obtain a delta value for the basic SoD strategy given a desired average transmission rate because it depends on the network performance, on the control system and on the nature of the sampled signal, which is, in most cases, unpredictable. A preliminary attempt to select dynamically a delta value for a simple control system is recently presented in [20], and, in this paper, we formulate the bases, improve the behavior, and extend the results for general signal tracking.

The paper is organized as follows. This first section was an introduction to present the general concepts of periodic sampling and send-on-delta (SoD) sampling. Section 2 studies the problems of selecting a delta value if a mean allowable bandwith is given for the basic SoD strategy. The algorithm to select dynamically a delta value is presented in detail in Section 3. Simulation results and comparative analysis between the proposed solution and a conformant periodic sampling strategy are presented in Section 4. Finally, in Section 5, the conclusions and future work are summarized.

## 2. Problem Statement

Usually, SoD strategies stand to reduce the amount of data that should be transmitted through a network to reduce resources. However, in some cases, the SoD strategy could be utilized by the senders to reduce the tracking error in place of reducing the amount of sent messages. In this work, we demonstrate that the tracking error generated by a basic SoD transmission strategy is in general smaller than the one generated by a conformant periodic transmission strategy.

The problem is that it is very difficult to prevent the throughput *λ* of an unpredictable signal x(t) given a fixed delta value *δ*, and, by extension, it is very difficult to obtain a fixed delta value given a mean throughput $\bar{\lambda }$.

From [17], the throughput *λ* of a monotonic continuous-time signal sampled periodically is given by the formula:

(2) $$\lambda =\frac{{\left|\dot{x}\right|}_{max}}{\varepsilon_{max}},$$

where ${\left|\dot{x}\right|}_{max}$ is the maximum of the absolute value of the signal time derivative and $\varepsilon_{max}$ is the upper bound of the signal tracking error.

For a basic SoD (with delta value *δ*) applied to a monotonic continuous signal, the upper bound of the signal tracking error matches the delta value. From [2], the upper and lower bounds of the basic SoD strategy are given by the Formula (3):

(3) $$\begin{array}{c} \lambda_{max}=\frac{\bar{\left|\dot{x}\right|}}{\delta },\\ \lambda_{min}=\frac{\bar{\left|\dot{x}\right|}}{\delta }-2\nu , \end{array}$$

where *ν* is the local extrema density, that is, the average number of signal peaks (maxima and minima) in a time unit, and $\bar{\left|\dot{x}\right|}$ is the average slope of the signal x(t) after an operation time of $T_{op}$, given by

$$\begin{array}{c} \bar{\left|\dot{x}\right|}=\frac{1}{T_{op}}\int_{0}^{T_{op}}\left|\dot{x}\left(\tau \right)\right|d\tau . \end{array}$$

If the signal x(t) is monotonously increasing or decreasing, the limits in Equation (3) match each other, and the relationship between *δ* and *λ* is fixed.

From Equation (3), given an average throughput $\bar{\lambda }$, the maximum and minimum values for *δ* are given by

(4) $$\begin{array}{c} \delta_{min}=\frac{\bar{\left|\dot{x}\right|}}{\bar{\lambda }},\\ \delta_{max}=\frac{\bar{\left|\dot{x}\right|}}{\bar{\lambda }+2\nu }. \end{array}$$

**Assumption** **1.** *From the Expression* (4)*, if during the test duration, the signal is monotonically increasing or decreasing, or has only a few maxima and minima per second compared to the average throughput (*$\nu \ll \bar{\lambda }$*), equality* (5) *holds*

(5) $$\delta =\frac{\bar{\left|\dot{x}\right|}}{\bar{\lambda }}.$$

**Proposition** **1.** Based on Assumption 1, the basic Send-on-Delta transmission strategy generates a smaller tracking error than a periodic transmission strategy with the same mean throughput.

**Proof.**  For a periodic transmission strategy, the average throughput matches the instantaneous throughput ($\bar{\lambda }=\lambda$); therefore, from Equations (2) and (5):

$$\frac{\delta }{\varepsilon_{max}}=\frac{\bar{\left|\dot{x}\right|}}{{\left|\dot{x}\right|}_{max}}.$$

From the properties of the expected value, it is always true that $\bar{\left|\dot{x}\right|}\leq {\left|\dot{x}\right|}_{max}$. Therefore,

(6) $$\delta \leq \varepsilon_{max}.$$

☐

This result can be extended to no-monotonous signals by dividing the signal into monotonous zones and maintaining the average throughput constant. If we define for the monotonous zone *i*:

- $\varepsilon_{max}^{i}$ as the upper bound in error tracking for the periodic sampling strategy, and
- $\delta^{i}$ as the upper bound in error tracking for the SoD sampling strategy,

then $\varepsilon_{max}^{i}\geq \delta^{i}\;\forall i$.

Intuitively, this means that the basic SoD transmission strategy has in general a smaller tracking error than the periodic transmission strategy for the same mean throughput.

### 2.1. Obtaining the Transmission-Rate Given a Delta Value.

Equation (5) presents an indeterminacy if δ=0 due to the continuous nature of the signal x(t). In practical implementations, discretized signals are used.

**Figure 1:**
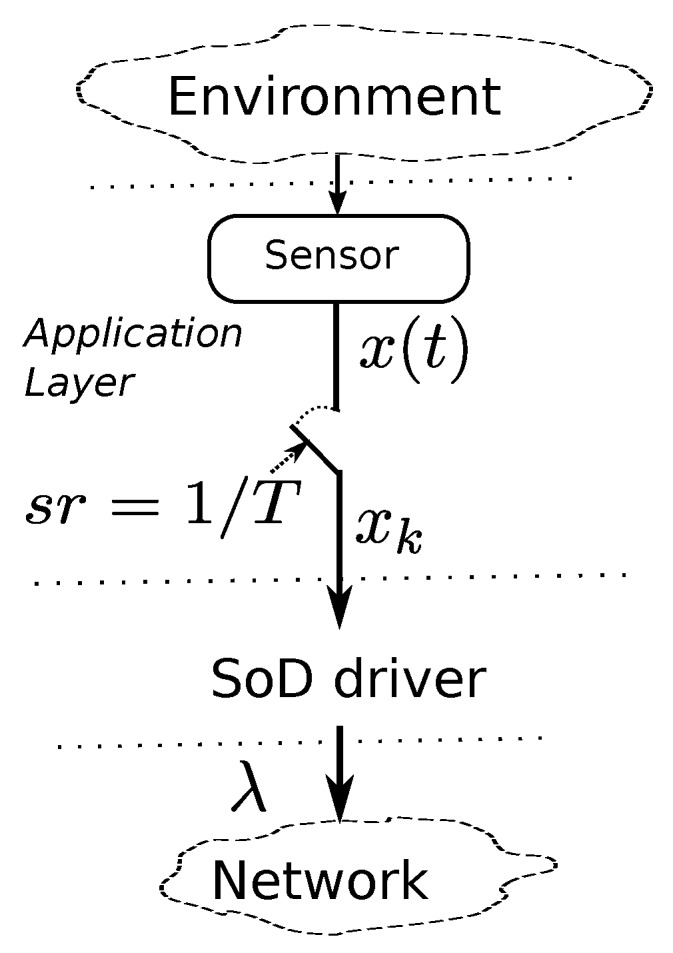
Discretized ($x_{k}$) raw signal (x(t)).

**Assumption** **2.** *Let us assume that the original raw signal* x(t) *is periodically discretized with sampling rate* sr=1/T *for computational use at the application layer. Samples are represented by* $x\left(kT\right)\equiv x_{k}$. *After that, the sample is delivered to the SoD driver that decides if it should be sent or not according to the send Function* (1). *If sent, a packet contains one sample. Figure 1 may help to visualize this assumption. At reception, the SoD driver uses the Zero Order Hold (ZOH) strategy, where the last received data is hold and delivered to the application layer until a new data arrives.*

Assumption 2 implies some consequences and clarifications:

- In contrast to the original sampling rate sr=1/T, we use the term *transmission-rate* or *throughput λ* (in packets per second) to define the amount of messages in a time unit that will be sent after the SoD strategy is applied by the SoD driver. Therefore, if there is not a SoD strategy implemented, the transmission and the sampling rates are equivalent (sr=λ).
- Assumption 2 allows for defining the normalized throughput $\lambda^{\prime }$ as the number of messages in a time unit divided by the original periodic sampling rate sr of the signal ($\lambda^{\prime }=\lambda /sr$). The normalized mean throughput $\bar{\lambda^{\prime }}$ is the mean throughput during the operation time, divided by sr ($\bar{\lambda^{\prime }}=\bar{\lambda }/sr$).
- During the operation time $T_{op}$, an amount of *N* samples are received by the SoD driver from the application layer. Therefore, $T_{op}=NT$.
- For a discretized signal $x_{k}\equiv x\left(kT\right)$, the value of $\bar{\left|\dot{x}\right|}$ after *N* samples is now
  $$\begin{array}{c} {\bar{\left|\dot{x}\right|}}_{N}=\frac{1}{N}\sum_{k=1}^{N}\frac{\left|x_{k}-x_{k-1}\right|}{t_{k}-t_{k-1}}=\frac{1}{NT}\sum_{k=1}^{N}\left|x_{k}-x_{k-1}\right|. \end{array}$$

Assumption 2 solves the indeterminacy of Equation (5) when δ=0 by imposing operational limits. If we define $\delta_{max}$ and $\delta_{min}$ as

(7) $$\begin{array}{c} \delta_{max}:=\max_{\begin{array}{c} 0\leq i<N\\ 0\leq j<N \end{array}}\left|x_{i}-x_{j}\right|,\\ \delta_{min}:=\min_{0\leq i<N}\left|x_{i}-x_{i+1}\right|, \end{array}$$

and define

(8) $$c_{N}=\frac{{\bar{\left|\dot{x}\right|}}_{N}}{sr}=T{\bar{\left|\dot{x}\right|}}_{N}=\frac{1}{N}\sum_{k=1}^{N}\left|x_{k}-x_{k-1}\right|,$$

then the relationship between *λ* and *δ* may be synthesized in Equation (9), where ${\bar{\lambda^{\prime }}}_{N}$ is the mean normalized throughput after sample *N*:

(9) $${\bar{\lambda^{\prime }}}_{N}=\left\{\begin{array}{cc} 0\; & \mathrm{if}\;\delta_{max}\;\leq \delta ,\\ \frac{c_{N}}{\delta }\; & \mathrm{if}\;\delta_{min}\leq \delta \leq \delta_{max},\\ 1\; & \mathrm{if}\;\delta <\delta_{min}. \end{array}\right.$$

### 2.2. Obtaining a Delta Value Given a Mean Available Transmission-Rate

From Equation (9), given a normalized available average transmission-rate ($\bar{\lambda_{a}^{\prime }}$) for an operation time $T_{op}=NT$ , $\delta =\delta_{N}$ can be expressed like:

(10) $$\delta_{N}=\left\{\begin{array}{cc} \delta_{min},\; & \mathrm{if}\;\frac{c_{N}}{\bar{\lambda_{a}^{\prime }}}\;\leq \delta_{min},\\ \frac{c_{N}}{\bar{\lambda_{a}^{\prime }}},\; & \mathrm{if}\;\delta_{min}\leq \frac{c_{N}}{\bar{\lambda_{a}^{\prime }}}\leq \delta_{max},\\ \delta_{max},\; & \mathrm{if}\;\delta_{max}<\frac{c_{N}}{\bar{\lambda_{a}^{\prime }}}. \end{array}\right.$$

## 3. Practical Implementation

The sampling rate sr=1/T at the application layer is known and the normalized available mean transmission rate $\bar{\lambda_{a}^{\prime }}$ is given as the desired final result. Conversely, x(t) and, consequently, $c_{N}$ changes in an unpredictable way and so happens with ${\bar{\lambda^{\prime }}}_{N}$ that may not follow $\bar{\lambda_{a}^{\prime }}$ at all. Therefore, $\delta_{N}$ should be calculated dynamically to achieve it. Relationship (10) is useful if the signal x(t) changes smoothly due to the average value of $c_{N}$, but the real nature of x(t) is unknown. However, some practical issues should be taken into account.

### 3.1. Practical *δ* Limits

It is unfeasible to know the values of $\delta_{min}$ and $\delta_{max}$ based on Equation (7). Therefore, some practical limits should be established. Taking into account that $\lambda_{max}$ is sr, and, therefore, $\lambda_{max}^{\prime }={\bar{\lambda^{\prime }}}_{max}=1$, the minimum value of *δ* is established as:

(11) $$\delta_{m}=c_{N}.$$

The maximum limit of *δ* can be established by a $\lambda_{min}$ value based on a maximum desired tracking error or on limits given by transparency or stability issues if the x(t) signal has a teleoperation or a control system nature. In these cases, the maximum value of *δ* is given by:

(12) $$\delta_{M}=\frac{c_{N}}{\lambda_{min}^{\prime }}.$$

### 3.2. Practical $\bar{\lambda_{a}^{\prime }}$ Implementation

The real average throughput after *N* samples (${\bar{\lambda }}_{N}$) is given by $M_{s_{N}}/NT$, where $M_{s_{N}}$ is the number of messages sent after an operation time of $T_{op}=NT$ time units. On the other side, the desired average throughput ($\bar{\lambda_{a}}$) could be given by $M_{a_{N}}/NT$, where $M_{a_{N}}$ is the allowed number of messages to be sent in $T_{op}$. That means that the sender has a quantity of messages to send ($M_{a_{N}}$) in an operation time ($T_{op}=NT$); However, since the operating time is not known in advance, the algorithm computations are performed in fixed time windows (ΔT).

**Assumption** **3.** *The whole operation time* $T_{op}$ *is an integer of* ΔT*:*

$$T_{op}=NT=\sum_{i=1}^{K}\Delta T=K\Delta T.$$

Assumption 3 may be fulfilled if ΔT is small enough compared to $T_{op}$ and eliminates unnecessary residual calculations.

Dividing calculations into *K* time windows, the average throughputs $\bar{\lambda_{N}}$ and $\bar{\lambda_{a}}$ may be replaced by the amount of messages in ΔT time units, that is:

(13) $$\begin{array}{c} \bar{\lambda_{N}}=\frac{M_{s_{N}}}{NT}=\frac{1}{K\Delta T}\sum_{i=1}^{K}M_{s_{i}},\\ \bar{\lambda_{a}}=\frac{M_{a_{N}}}{NT}=\frac{1}{K\Delta T}\sum_{i=1}^{K}M_{a_{\Delta T}}=\frac{M_{a_{\Delta T}}}{\Delta T}, \end{array}$$

where $M_{s_{i}}$ is the amount of sent messages at the end of the *i*-th time-window and $M_{a_{\Delta T}}$ the amount of allowed messages to be sent in ΔT. Since $\bar{\lambda_{a}}$ and ΔT are constants, so is $M_{a_{\Delta T}}$. Therefore, $\bar{\lambda_{a}^{\prime }}$ is replaced by:

$$\begin{array}{c} \bar{\lambda_{a}^{\prime }}=\frac{T\cdot M_{a_{\Delta T}}}{\Delta T}. \end{array}$$

**Definition** **1.** *The remaining number of messages during the i-th time-window* $M_{r_{i}}\left(t\right)$ *is the difference between: the real number of available messages to be sent at the start of the i-th time-window* $M_{a_{i}}$*, and the number of sent messages during the i-th time-window* $M_{s_{i}}\left(t\right)$*:*

(14) $$M_{r_{i}}\left(t\right)=M_{a_{i}}-M_{s_{i}}\left(t\right).$$

The real available number of messages to be sent $M_{a_{i}}$ will be defined below.

**Definition** **2.** *The container number of messages at the end of the i-th time-window* $M_{c_{i}}$ *is the sum of:* $M_{r_{i}}$ *at the end of the i-th time-window, and the remaining number of messages of previous time-windows:*

(15) $$M_{c_{i}}=\sum_{k=1}^{i}M_{r_{k}}\left(\Delta T\right).$$

**Definition** **3.** *The real available number of messages to be sent at the start of the i-th time-window* $M_{a_{i}}$ *is the sum of:* $M_{a_{\Delta T}}$ *and* $M_{c_{i-1}}$*:*

(16) $$M_{a_{i}}=M_{a_{\Delta T}}+M_{c_{i-1}}.$$

Previous definitions have some remarks:

- t=0 at the start and t=ΔT at the end of each time-window, that is, t∈[0,ΔT]. Mathematically, t=NTmodΔT, where *N* is the application sample number and *T* is the application sampling period (see Assumption 2).
- for the first time-window (i=1), $M_{c_{0}}=0$ and, therefore, $M_{a_{1}}=M_{a_{\Delta T}}$.
- $M_{r_{i}}$ may have a negative value if $M_{s_{i}}>M_{a_{i}}$. Therefore, $M_{c_{i}}$ may also have a negative value. If $M_{c_{i}}>0$ there is *credit*, that is, the sender can send more than the available number of messages and, if $M_{c_{i}}<0$, there is *debt*, that is, the sender should send if possible less than $M_{a_{i}}$ messages.

From Equations (14) and (16),

(17) $$M_{r_{i}}\left(t\right)=M_{a_{\Delta T}}-M_{s_{i}}\left(t\right)+M_{c_{i-1}}.$$

**Remark** **1.** *If, at the end of the operation time,* $M_{c_{i}}$ *tends to 0, that is,*

$${\left.\sum_{i=1}^{K}M_{r_{i}}\right|}_{K\Delta T\to T_{op}}\to 0,$$

*then*

(18) $$\bar{\lambda_{a}}=\sum_{i=1}^{K}\bar{\lambda_{a_{i}}},$$

*where* $\bar{\lambda_{a_{i}}}=M_{a_{i}}/\Delta T$.

Taking into account Remark 1 and the central term of Equation (10), during the *i*-th time window, the delta value is calculated in real-time as:

(19) $$\delta_{N}=\delta_{i}\left(t\right)=\frac{\Delta T-t}{T\cdot M_{r_{i}}\left(t\right)}c_{N},$$

where *N* matches NT=(i−1)ΔT+t.

In practical implementations, it could be recommended that the container messages would not be available to be spent at the start of the time-window, because, in these cases, the δ(t) calculations may oscillate too much from one window to the other.

To solve this practical issue, we propose a procedure to calculate $M_{r_{i}}\left(t\right)$ presented below, represented as:

(20) $$M_{r_{i}}\left(t\right)=M_{a_{\Delta T}}-M_{s}\left(t\right)+f\left(M_{c_{i-1}},M_{s}\left(t\right)\right),$$

where $f(M_{c_{i-1}},M_{s}\left(t\right))$ is a function of the container messages updated every time a new message is sent.

The procedure to calculate $M_{r_{i}}\left(t\right)$ is presented in *Procedure* 1.

Basically, for every time-window at the instant when a new message is sent, the procedure:

- does not decrease $M_{r_{i}}$ if there is still a positive value in the container (*credit*, that is $M_{c_{i}}>0$) and this decreases the container, or
- decreases $M_{r_{i}}$ twice if there is a negative value in the container (*debt*, that is $M_{c_{i}}<0$) and increases the container, or
- decreases $M_{r_{i}}$ once if there is 0 in the container ($M_{c_{i}}=0$).

The result of the procedure is replaced in Equation (19). Although $M_{r_{i}}\left(t\right)$ may become 0 or a negative value, it does not affect the $\delta_{N}$ results due to the limits in Equation (10).
**Procedure 1** Calculate $M_{r}$$M_{r}=M_{a_{\Delta T}}$$M_{s}=0$$M_{c}=0$t=0*MessSent*:**if** Message sent **then**    $M_{s}\leftarrow M_{s}+1$    $M_{r}\leftarrow M_{r}-1$// remaining messages decreases    **if**
$M_{c}>0$
**then**// if there are *credit*        $M_{r}\leftarrow M_{r}+1$// remaining messages increases        $M_{c}\leftarrow M_{c}-1$// *credit* decreases        **goto**
*NewWin*.    **else if**
$M_{c}<0$
**then**// if there are *debt*        $M_{r}\leftarrow M_{r}-1$// remaining messages decreases        $M_{c}\leftarrow M_{c}+1$// *debt* decreases*NewWin*:**if**
t≥ΔT
**then**// A new time-window starts    $M_{c}\leftarrow M_{c}+M_{r}$    $M_{r}=M_{a_{\Delta T}}$    $M_{s}=0$    t=0**goto**
*MessSent*.


### 3.3. Other Practical Issues

Equation (8) shows that, at the start of the algorithm, the delta value may change too fast following $c_{N}$ when *N* is small. If, at the start of the algorithm, the signal is in a stationary state for $N_{0}$ samples, the $c_{N_{0}}$ value is small. Therefore, Equation (8) may be rewritten as:

(21) $$c_{N}=\frac{1}{N_{0}+N}\left(c_{N_{0}}+\sum_{k=1}^{N}\left|x_{k}-x_{k-1}\right|\right).$$

This solution smooths the effect that could happen if, at the start of the algorithm, the signal suddenly changes very fast and δ(t) grows accordingly.

Another issue is that, from Equation (19), if the $c_{N}$ variable grows too much, or if the $M_{r_{i}}\left(t\right)$ variable becomes too small (that is, if the predicted amount of data allowable to be sent is spent before the time-window is finished), it is important to apply upper bounds for the delta value. In this case, if the delta value exceeds the $\delta_{M}$ limit defined in Equation (12), it is reset to the average value of *δ* in the *N*-th algorithm implementation given by:

(22) $$\bar{\delta_{N-1}}=\frac{1}{N-1}\sum_{k=1}^{N-1}\delta_{k}.$$

In the same way, at the end of the time-window (t=ΔT), from Equation (19), the value of δ(t) may become too small if there are no messages sent (or too few). In this case, if the delta value is below the $\delta_{m}$ limit defined in Equation (11), it is also reset to $\bar{\delta_{N}}$.

### 3.4. Summarizing the Algorithm

Expression (23) summarizes the proposed algorithm, where $c_{N}$ is given finally by Equation (21):

(23) $$\delta_{N}=\left\{\begin{array}{cc} \bar{\delta_{N-1}},\; & \mathrm{if}\;\;\frac{\Delta T-t}{T\cdot M_{r_{i}}\left(t\right)}c_{N}\;\leq \delta_{m},\\ \frac{\Delta T-t}{T\cdot M_{r_{i}}\left(t\right)}c_{N},\; & \mathrm{if}\;\delta_{m}\leq \frac{\Delta T-t}{T\cdot M_{r_{i}}\left(t\right)}c_{N}\leq \delta_{M},\\ \bar{\delta_{N-1}},\; & \mathrm{if}\;\delta_{M}<\frac{\Delta T-t}{T\cdot M_{r_{i}}\left(t\right)}c_{N}, \end{array}\right.$$

where t∈(0,ΔT), $c_{N}$ is calculated in Equation (21), $\bar{\delta_{N-1}}$ is calculated in Equation (22) and $M_{r_{i}}\left(t\right)$ is calculated applying the Procedure 1.

**Figure 2:**
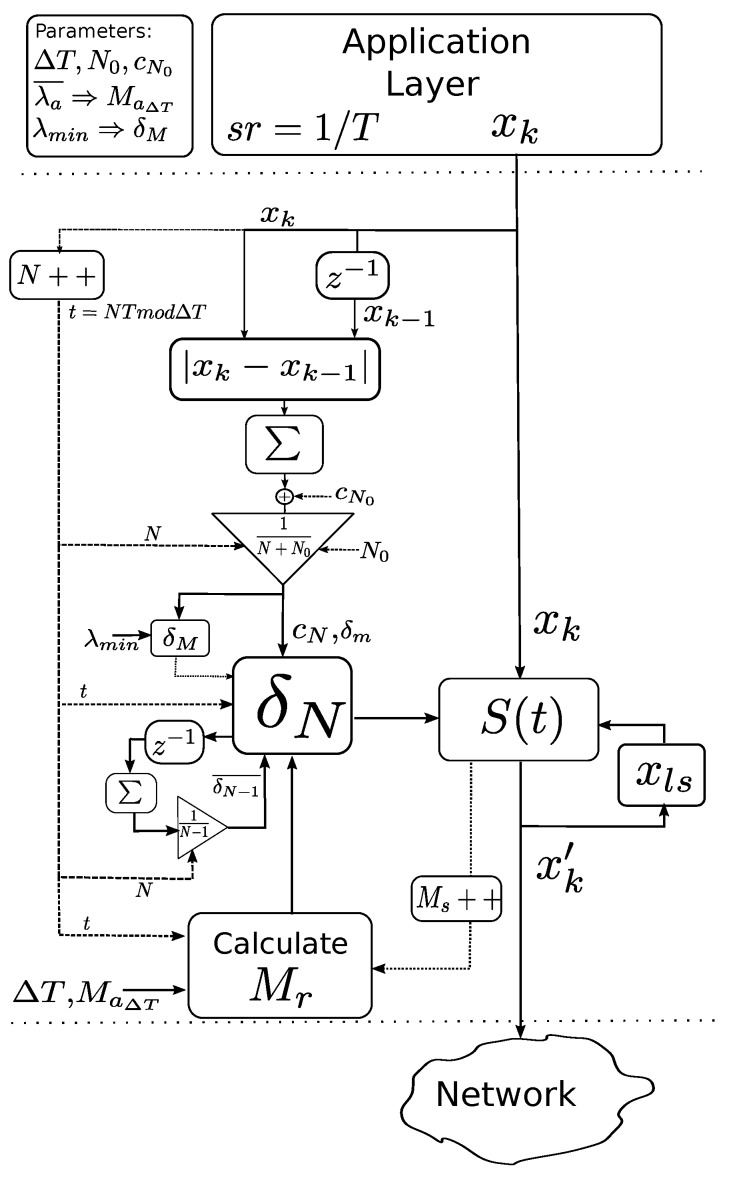
$\delta_{N}$ algorithm.

A schematic about the whole algorithm is illustrated in Figure 2.

## 4. Simulation

Simulations are performed with two main objectives: on the one side, the results of the simulations have to show that the algorithm gets the desired mean available transmission rate $\bar{\lambda_{a}}$ for tested signals, and, on the other side, the proposed algorithm should feature a smaller signal tracking error at reception than a conformant periodic sampling strategy.

Simulations are applied to three test signals. The signals are selected according to the unit step responses for a second-order control system following Equation (24) with A=1, ϕ=π/2 and different *ρ* and *μ* parameters. Parameter *ρ* delimits the asymptotic contention of the overshoot and undershoot, and *μ* delimits the number of oscillations before the settling time. For simplicity, we set *ρ* to 1 (ρ=1) for the three signals:

(24) $$x\left(t\right)=A\left(1-e^{\rho t}\frac{sin(\mu t+\phi )}{sin(\phi )}\right).$$

The first signal is an over-damped step-response, the second one is an under-damped step-response and the last one is also an under-damped step response but with a high-oscillating behaviour. The nature of the signals may have an important effect on the results, reflected not only by the $c_{N}$ variable but in the number of sent messages ($M_{s}$) in the time-window. For example, for a signal that changes rapidly during a short period but remains constant during a long period, the proposed SoD sampling should perform significantly better than the periodic one; on the contrary, for a signal that changes during the whole operation time, the proposed SoD sampling should perform worse than before, but, in any case, based on Proposition 1, it has to perform better than the periodic one if the delta value *δ* is well selected to match the desired throughput.

To observe the algorithm behavior in a long-time operation, the signals are periodically repeated, using a repetition period of $T_{r}=20$ s for the main tests. At the end of this section, some results with different repetition periods are presented to observe the behaviour for different kinds of signals.

Simulations are performed using the network simulator NS2. Both extremes are connected through a packet switched network with a bandwith of 10 Mbps and 1 ms delay between hosts. There is no residual traffic in the network. We consider that the available transmission rate for each sender is of 10 kbps and that the senders may take advantage of the whole available transmission rate over 1000 s. The raw original signal is sampled at 1000 pkt/s. We consider that the packet contains residual information (control or other data) in addition to the sensed data. Therefore, the packet size is 125 bytes and the available transmission rate is 10 pkt/s. The simulation parameters are presented in Table 1.

**Figure 3:**
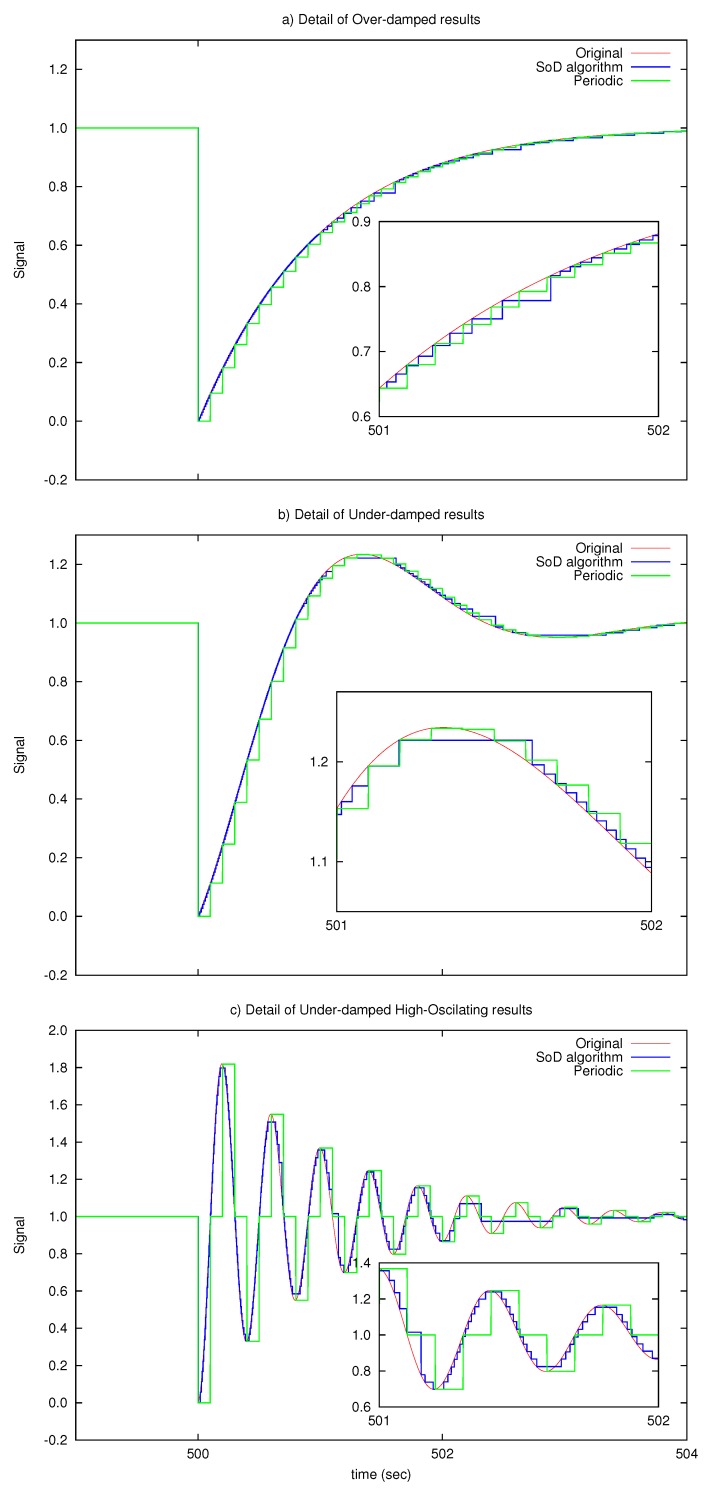
Detail of transmission results for: (**a**) over-damped signal; (**b**) under-damped signal; (**c**) under-damped High-Oscillating signal.

Figure 3 illustrates a detail of the sent signals comparing the periodic transmission with the proposed SoD transmission strategy for $\bar{\lambda_{a}}=10$ kbps. The graph results show that the proposed SoD algorithm tracks in a more accurate way the original signal for the three proposed test signals.

**Figure 4:**
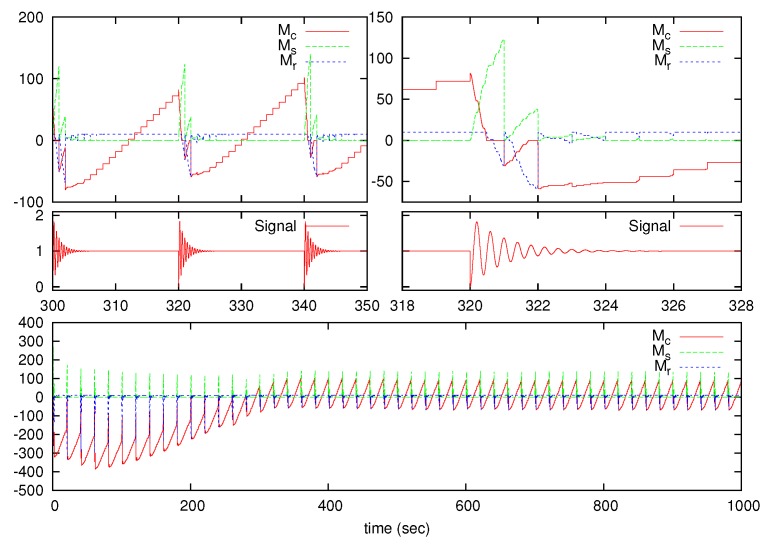
Algorithm behavior for the under-damped High-Oscillating signal. Details for the $M_{s}$, $M_{r}$ and $M_{c}$ variables.

**Figure 5:**
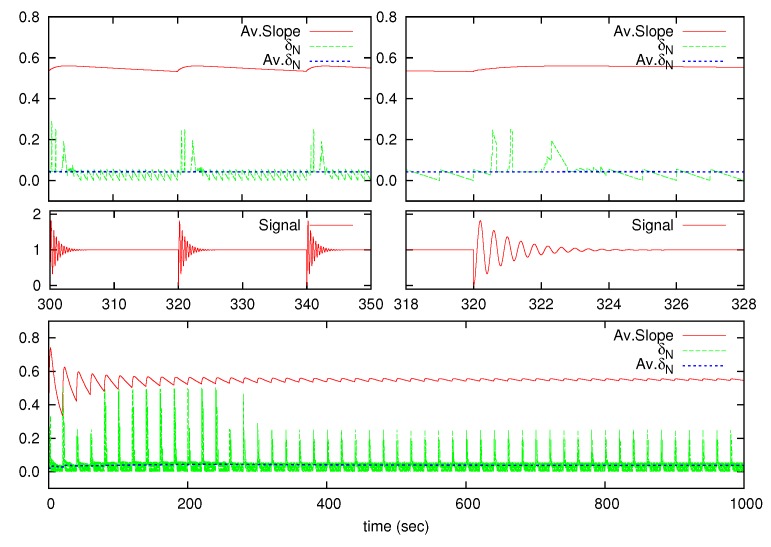
Algorithm behavior for the under-damped High-Oscillating signal. Details for the ${\bar{\left|\dot{x}\right|}}_{N}$, $\delta_{N}$ and ${\bar{\delta }}_{N}$ variables.

The algorithm behavior is shown in Figure 4 and Figure 5 using the under-damped High-Oscillating test signal, which was the worst case. Figure 4 shows the results from Procedure 1 to calculate $M_{r}$, where variables $M_{s}$ and $M_{c}$ are also drawn. As seen, $M_{c}$ converges to 0 for long-time operation. Figure 5 shows the ${\bar{\left|\dot{x}\right|}}_{N}$, $\delta_{N}$ and ${\bar{\delta }}_{N}$ variables. Similar results are achieved for the over-damped and under-damped signals.

**Figure 6:**
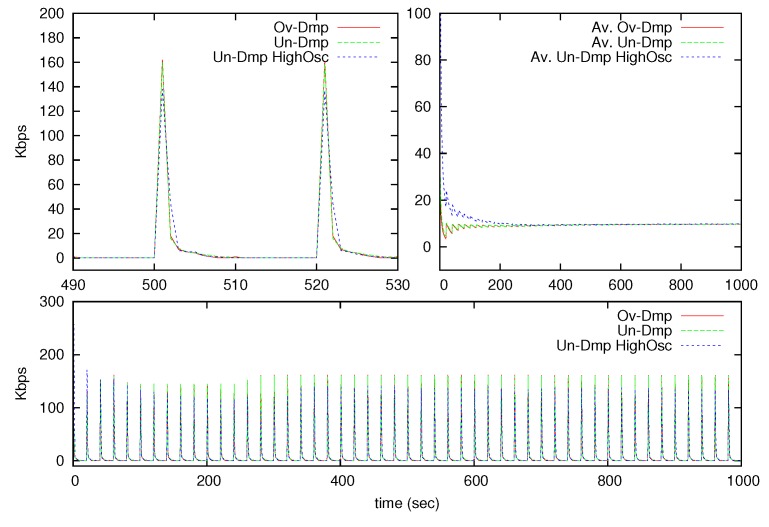
Throughput behavior for the three signals in Kbps.

**Figure 7:**
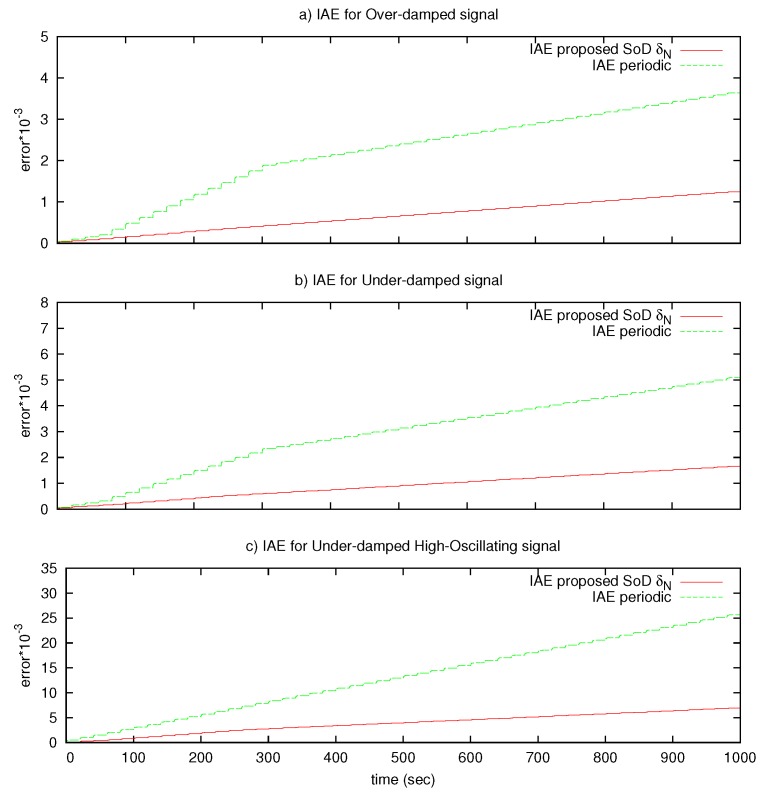
Integral Absolute Error (IAE) results of the proposed algorithm and the conformant periodic transmission for the three signals.

The throughput and the error are illustrated in Figure 6 and Figure 7. The bottom of Figure 6 shows the throughput behavior during the whole testing time; the upper left corner shows a detail at the middle of the testing time and finally the upper right corner shows the average value of the throughput during the entire testing duration. As expected, the throughput is concentrated at the moments where the signal changes faster, but the average throughput value converges to the desired average throughput in long-time operations as seen in the upper right corner of the figure.

The tracking error is calculated comparing the original signal with the transmitted signal for the two transmission strategies: the proposed SoD strategy and the conformant periodic transmission strategy. That is, for the SoD strategy, the error is given by Expression (25), and, for the periodic sampling strategy, the error is given by Expression (26):

(25) $$e\left(t\right)=∥x_{1}{\left(t\right)}_{\delta =0}-x_{1}{\left(t\right)}_{\delta }∥,$$

(26) $$e\left(t\right)=∥x_{1}{\left(t\right)}_{sr=1000}-x_{1}{\left(t\right)}_{sr=10}∥.$$

Note that $x_{1}{\left(t\right)}_{\delta =0}=x_{1}{\left(t\right)}_{sr=1000}$ represents the original signal, and $x_{1}{\left(t\right)}_{\delta }$ stands for the transmitted signal after applying the SoD driver.

In addition, the Integral Absolute Error ($IAE\left(\delta \right)=\int_{0}^{T_{t}}\left|e\left(\tau \right)\right|d\tau$) is measured and presented in Figure 7. This figure shows that the error measured using the SoD algorithm is much smaller than the conformant periodic transmission strategy in long-time simulation intervals for the three tested signals.

Despite the graphs showing the effectiveness of the proposed algorithm, some numerical results are given in Table 2.

**Figure 8:**
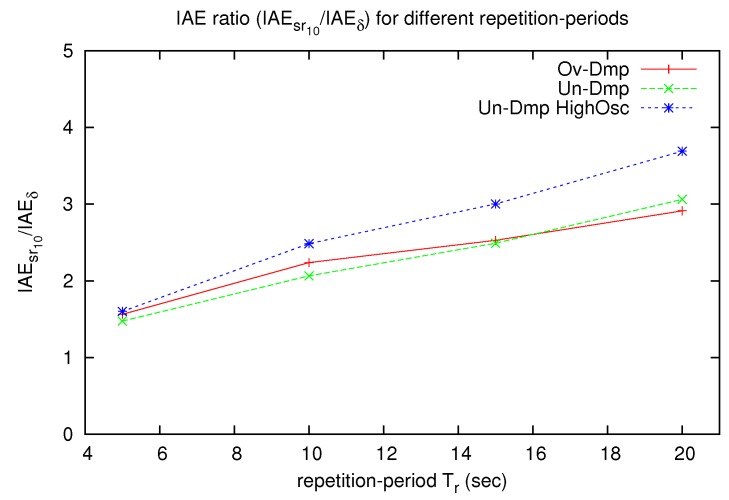
IAE ratio ($IAE_{sr_{10}}/IAE_{\delta }$) between the conformant periodic sampling and the SoD sampling for different repetition periods.

Numerical results are calculated for the three signals with a repetition period $T_{r}$ of 20 s. These results reveal that the proposed algorithm performs well in long-time operation, and that the tracking error is much smaller than the conformant periodic transmission strategy. However, as mentioned at the start of this section, the nature of the test signals may affect the results, particularly the error results, mainly if the signal spends a lot of time without changes. Therefore, Figure 8 presents an error comparison for the three signals with $T_{r}\in \left\{5,10,15,20\right\}$ s. The vertical axis represents the ratio between the measured IAE of the conformant periodic sampling and the proposed SoD sampling ($IAE_{sr_{10}}/IAE_{\delta }$).

In all of the cases, the proposed SoD sampling strategy performs better than the conformant periodic sampling. Besides that for shorter repetition periods, the measured IAE for both strategies are closer, the IAE for the periodic one is at least 1.5 times bigger than the proposed SoD, as expected based on Proposition 1.

In spite of these results, the real throughput is difficult to adjust to match exactly the desired throughput, especially at the start of the algorithm. Nevertheless, the results are satisfactory in long-time operation for the three signals, matching more than 90% of the desired throughput 200 s after the start and matching more than 96% of the desired throughput at the end of the test times. These results are very similar for the other repetition periods.

## 5. Conclusions

This paper presents an algorithm to reduce the signal tracking error taking advantage of the basic Send-on-Delta (SoD) sampling strategy. Given a mean available bandwith, the paper demonstrates that, for continuous signals, the SoD sampling strategy performs better than a conformant (same available bandwith) periodic transmission. The algorithm is tested for three signals based on usual step responses of second order control systems, and is then compared with the periodic transmission. The tests compare two indicators: the tracking-error and the average-throughput. Results show that the tracking-error is definitely smaller with the proposed method and that the average-throughput is achieved in long-time operation. The fact that the average throughput is achieved in long-time operation is due to the proper nature of the SoD technique, where samples are concentrated when the signal changes faster; therefore, the throughput can not be uniform and has to be analyzed as an average throughput in time, with more accurate results the longer the operation-time.

The results invite us to improve the algorithm behavior basically by minimizing the convergence time of the mean throughput to the desired one. This could be achieved by changing some initial parameters, parameters that are actually very few, to simplify the algorithm. Other SoD techniques, such as the linear predicted SoD technique that could be very interesting for continuous signal tracking as seen in [18], are being studied in order to also develop an algorithm to reduce the tracking-error given a mean available transmission rate.

Finally, one of the main interests in developing the algorithm is to dynamically determine the delta value to reduce the error in place of reducing the throughput if the available transmission rate is dynamically shared by different senders—for example, for distributed sensor networks, multi-agent systems or distributed systems in general.

## Figures and Tables

**Table 1:** Simulation parameters.

Test Signals $x\left(t\right)=(1-e^{t}\sin\left(\mu t+\pi /2\right))$		
Over-damped	Under-damped	Under-damped High-Oscil.
μ=0.25	μ=2	μ=16
**Network Loop**		
Bandwith = 10 Mbps	τ=1 ms	Full Duplex Queue size = 2
**Algorithm Parameters**		
$\bar{\lambda_{a}}=10$ kbps	sr=1000 pkt/s	$\lambda_{min}=1$ kbps
$pkt_{size}=125$ Bytes	T=0.001 s	
$N_{0}=10^{4}$	ΔT=1 s	$T_{oper}=1000$ s
$c_{N_{0}}=0.0$	$M_{a_{\Delta T}}=10$ pkts	

**Table 2:** Results summary.

Proposed SoD δ(t) Algorithm			
	Over-Damped	Under-Damped	UD High Oscill
Average throughput (${\bar{\lambda }}_{N}$)	9,627 bps	9,634 bps	9,687 bps
Average *δ*	0.006538	0.008712	0.037178
IAE	1,249	1,660	6,946
**Conforming Periodic Transmission**			
	Over-Damped	Under-Damped	UD High Oscill
Used transmission-rate	10 kbps	10 kbps	10 kbps
IAE	3,637	5,083	25,628
